# [Retracted] Reduction of miR‑29a‑3p induced cardiac ischemia reperfusion injury in mice via targeting Bax

**DOI:** 10.3892/etm.2024.12771

**Published:** 2024-11-25

**Authors:** Liang Zhang, Jian Zhang, Qiguang Tong, Guannan Wang, Hongling Dong, Zhonglu Wang, Qi Sun, Hangyu Wu

Exp Ther Med 18:1729–1737, 2019; DOI: 10.3892/etm.2019.7722

Following the publication of this paper, it was drawn to the Editors’ attention by a concerned reader that the Transwell cell flow cytometric assay data shown in Fig. 2B on p. 1733 were strikingly similar to data appearing in different form in a pair of other articles written by different authors at different research institutes that had already been published elsewhere prior to the submission of this paper to *Experimental and Therapeutic Medicine*. In view of the fact that the abovementioned data had already apparently been published previously, the Editor of *Experimental and Therapeutic Medicine* has decided that this paper should be retracted from the Journal. The authors were asked for an explanation to account for these concerns, but the Editorial Office did not receive a reply. The Editor apologizes to the readership for any inconvenience caused.

